# Insect cuticular proteins and their role in transmission of phytoviruses

**DOI:** 10.1016/j.coviro.2018.07.015

**Published:** 2018-12

**Authors:** Maëlle Deshoux, Baptiste Monsion, Marilyne Uzest

**Affiliations:** BGPI, Univ Montpellier, CIRAD, INRA, Montpellier SupAgro, Montpellier, France

## Abstract

Many viruses of agricultural importance are transmitted to host plants via insect vectors. Characterizing virus–vector interactions at the molecular level is essential if we are to fully understand the transmission mechanisms involved and develop new strategies to control viral spread. Hitherto, insect proteins involved in virus transmission have been characterized only poorly. Recent advances in this topic, however, have significantly filled this knowledge gap. Among the vector molecules identified, cuticular proteins have emerged as key molecules for plant virus transmission, regardless of transmission mode or vector considered. Here, we review recent evidence highlighting that the CPR family, and particularly RR-1 proteins, undoubtedly deserves special attention.

## Highlights

• Cuticular proteins play key roles in plant virus transmission.
• RR-1 and RR-2 are the main cuticular proteins involved in virus–vector interactions.
• RR-1 protein is involved in transmission of a noncirculative virus.
• RR-1 protein is involved in transmission of a circulative virus.
• The role of other cuticular proteins in virus transmission is poorly characterized.

## Graphical abstract

**Figure:**
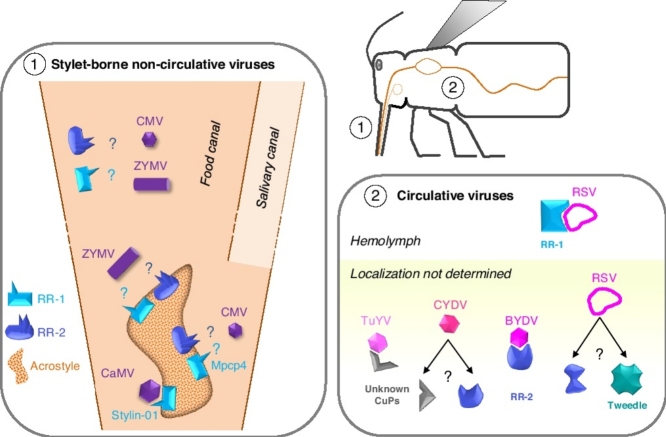

**Current Opinion in Virology** 2018, **33**:137–143

This review comes from a themed issue on **Virus-vector interactions**

Edited by **Anna E Whitfield** and **Ralf G Dietzgen**

For a complete overview see the Issue and the Editorial

Available online 20th September 2018

**https://doi.org/10.1016/j.coviro.2018.07.015**

1879-6257/© 2018 The Authors. Published by Elsevier B.V. This is an open access article under the CC BY license (http://creativecommons.org/licenses/by/4.0/).

## Introduction

To ensure sustainability in the environment, phytoviruses must overcome two major constraints: their hosts are immobile, and the plant cell wall represents a physical barrier that viruses have to cross before they can replicate and spread in plant tissues. Most plant viruses are transmitted horizontally and re-transported by plant-feeding organisms (vectors) that are able to move from plant to plant [1,2]. The most frequent vectors of plant viruses are hemipteran and thysanopteran insects with piercing-sucking mouthparts, including aphids, whiteflies, leafhoppers, planthoppers, and thrips [2]. Virus–vector interactions, which are sophisticated and highly specific [3], can be classified into two main categories (for a review see [4]). Noncirculative viruses are reversibly attached to the cuticle of the insect mouthparts, in the stylets or foregut of their vectors [4, 5, 6], during their journey from one plant to another. Circulative viruses are ‘internalized’ in the vector body, and must cross the gut barrier to reach the hemolymph and/or other tissues. Ultimately, viruses reach the salivary glands, and are injected, together with vector saliva, into new host plants. A few virus species have been shown to replicate within their vectors during their journey and have been classified in the circulative propagative subcategory [7]. Numerous studies have focused on elucidating the mechanisms underlying vector transmission [3,5,6,8], and viral determinants have been well characterized for most plant virus species studied. These include structural proteins, membrane viral glycoproteins, or non-structural virus-encoded proteins [4,9,10]. However, an extensive study of virus–vector interactions at the molecular level is still challenged by the difficulty of identifying vector partners and validating their role in virus transmission [11^••^,12^••^,13^••^,14^••^,15^••^].

Nonetheless, considerable efforts have been made to develop complementary approaches and high-throughput methods to help identify vector proteins involved in virus–vector interactions. Among these interacting molecules are several cuticular proteins (CuPs) [13^••^,15^••^,^16^,^17^,18^••^,^19^, ^20^, ^21^, ^22^, ^23^, ^24^, ^25^, ^26^]. CuPs are chitin-binding proteins that contribute to cuticle structural integrity, and reflect its diversity and mechanical properties [27, 28, 29]. CuPs have been classified into 14 families [30^•^,^31^,^32^], the most abundant by far being the CPR family comprising proteins with a Rebers and Riddiford (RR) consensus [33]. The RR family is divided into three subfamilies, RR-1, RR-2, RR-3 [30^•^], to which most identified virus-interacting proteins can be assigned (Table 1). Their role as a key partner of both noncirculative and circulative plant viruses was hitherto unforeseen. Here, we review striking advances in the characterization of CuP–virus interactions that have brought novel insights to the field of vector transmission of plant viruses.

**Table 1:** Cuticular proteins (CuPs) identified in the virus–insect vector interaction studies presented in this review

Virus species/genus	Vector — *species*	Transmission mode	CuPs identifier^a^ — *other name*	Protein family^b^ (subfamily)	Approaches	Reference
ZYMV/*Potyvirus*	Aphid **—** *M. persicae*	Noncirculative	AAO63549	CPR (RR-2)	Urea extraction of aphid CuPs, 1-D & 2-D gel electrophoresis, Far-western blot, MS analyses	[17]
			AAL29466	CPR (RR-2)		
			AAZ20451	CPR (RR-1)		
			AAZ20447	CPR (RR-2)		
CaMV/*Caulimovirus*	Aphid	Noncirculative	ND	ND	Biochemical characterization, Stylet immunolabeling	[40^•^]
CaMV/*Caulimovirus*	Aphid **—** *A. pisum, M. persicae*	Noncirculative	MG188739 **—** *Stylin-01*	CPR (RR-1)	Stylet immunolabeling, Colocalization, *in vitro* competition assays, RNAi	[15^••^]
			MG188741 **—** *Stylin-01*	CPR (RR-1)		
CMV/*Cucumovirus*	Aphid **—** *M. persicae*	Noncirculative	DQ108938 **—** *Mpcp4*	CPR (RR-1)	YTH	[22]
CMV/*Cucumovirus*	Aphid **—** *A. pisum*	Noncirculative	ND	CPR (RR-2)	Peptide array (RR-2 proteins)	[25]
TuYV^c^/*Polerovirus*	Aphid **—** *M. persicae*	Circulative	ND^d^	ND	Whole cell lysate (aphids), 1-D & 2-D gel electrophoresis, Far-western blot & MS analyses	[16]
CYDV-RPV/*Polerovirus*	Aphid **—** *S. graminum*	Circulative	gi:193647865	CPR (RR-2)	Genetics coupled to 2-D-DIGE & MS analyses	[18^••^]
			gi:193706873	ND^d^		
			gi:193647875	CPR (RR-2)		
			gi:193582403	CPR (RR-2)		
BYDV-GPV/*Luteoviridae*^e^	Aphid **—** *R. padi*	Circulative	gi:288558725 **—** *Cp 62 precursor*	CPR (RR-2)	iTRAQ & MS analyses	[20]
			NP_001156154.1 **—** *Cp 5 precursor*	CPR (RR-2)		
RSV/*Tenuivirus*	Planthopper **—** *L. striatellus*	Circulative-Propagative	KC485263 **—** *CPR1*	CPR (RR-1)	YTH, Chemiluminescent co-IP, Colocalization, GST pull-down, RNAi	[13^••^]
RSV/*Tenuivirus*	Planthopper **—** *L. striatellus*	Circulative-Propagative	XM_014390248.1 **—** *Cuticle protein A3A like*	CPR (RR-2)	YTH	[26]
			JAS02196.1	Tweedle		

a Given accession numbers from original studies.

b Classified using CutProtFam-Pred (http://aias.biol.uoa.gr/CutProtFam-Pred/).

c Formerly BWYV-FL1 (beet western yellows virus).

d published accession number does not correspond to a CuP according to databases and CutProtFam-Pred.

e Unassigned member in the family Luteoviridae. CuP: cuticular protein; ND: not determined; MS: mass spectrometry; RNAi: RNA interference; DIGE: difference gel electrophoresis; iTRAQ: isobaric tags for relative and absolute quantification; co-IP: co-immunoprecipitation; YTH: yeast two-hybrid. ZYMV: zucchini mosaic virus; CaMV: cauliflower mosaic virus; CMV: cucumber mosaic virus; TuYV: turnip yellows virus; CYDV: cereal yellow dwarf virus; BYDV: barley yellow dwarf virus, RSV: rice stripe virus.

### Role of cuticular proteins in noncirculative plant virus transmission

Noncirculative viruses bind reversibly to specific retention sites on the cuticle of the feeding apparatus. Therefore, virus-interacting molecules should be cuticular compounds that fulfill the role of virus receptors. To date, receptors of foregut-borne viruses have been poorly characterized [34,35], and no CuP has been shown to be involved in their retention or transmission. The great majority of noncirculative viruses, among which are members of the families *Potyviridae*, *Bromoviridae* and *Caulimoviridae*, is retained in the stylets [36, 37, 38], whose composition is poorly characterized [39]. Using *in vitro* interaction assays on dissected stylets, Uzest *et al.* [38] demonstrated that the receptor of the cauliflower mosaic virus (CaMV) was a CuP located at the fused food/salivary common canal of aphid maxillary stylets, on the acrostyle — an organ discovered later [40^•^] in an area described to harbor receptors of potyviruses and cucumoviruses [37,41]. However, binding to the acrostyle has been demonstrated only for a caulimovirus, and direct evidence of virus retention within the common canal is still lacking for other viruses.

**Figure 1:**
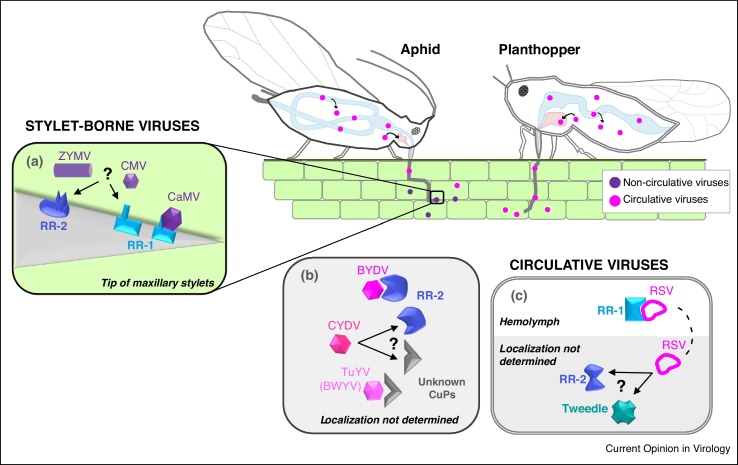
Plant virus–cuticular protein (CuP) interactions. **(a)** Interaction of noncirculative viruses with cuticular proteins at the tip of aphid maxillary stylets: cauliflower mosaic virus (CaMV) with RR-1 protein via its helper protein P2 [15^••^], cucumber mosaic virus (CMV) with RR-1 and/or RR-2 proteins [22,25], zucchini yellow mosaic virus (ZYMV) via its helper protein HC-Pro with RR-1 and/or RR-2 proteins [17]. **(b)** Interaction of barley yellow dwarf virus (BYDV), cereal yellow dwarf virus (CYDV) and turnip yellow virus (TuYV), three circulative viruses, with RR-2 and/or unknown cuticular proteins within their aphid-vector body [16,18^••^,^20^]. **(c)** The nucleocapsid protein of the circulative rice stripe virus (RSV) binds an RR-1 protein in the hemolymph of its planthopper vector [13^••^], and may also interact with another RR-2 and a Tweedle cuticular protein [26].

Dombrovsky *et al.* [17] were the first to identify cuticular partners of a noncirculative virus, the zucchini yellow mosaic virus (ZYMV) — a potyvirus that interacts with its aphid vectors through a viral-encoded protein (helper component, HC-Pro [42]). A far-western blot approach allowed detection of a few spots that specifically interacted with wt ZYMV-HC-Pro combinations, but not with a transmission-defective mutant. Out of nine spots microsequenced, four were identified as CuPs: one RR-1 and three RR-2 proteins (Table 1). At the time, it was not possible to ascertain the presence of any of these CuPs in the stylets, nor to confirm the biological relevance of these results. However, since then, using immunodetection approaches, some peptides have been identified at the tip of maxillary stylets [15^••^,^25^,40^•^], among which the peptide PepS was found to be present in two CuPs identified in this study (AAO63549.1 and AAL29466.1), reinstating them as prime candidate receptors of ZYMV (Figure 1). Further investigations should determine if ZYMV is retained in the common canal, and evaluate the role of these RR-2 proteins in ZYMV transmission.

Two independent studies on cucumber mosaic virus (CMV) receptor candidates were published in 2017 [22,25]. This cucumovirus, transmitted by aphids, interacts directly with vector receptors through its coat protein [43,44]. In the first study, using a yeast-two-hybrid (YTH) system, Liang and Gao [22] reported interaction between the coat protein of CMV, and one of four reported *Myzus persicae* CuPs [45], the RR-1 protein Mpcp4 (Table 1, Figure 1). Whether this interaction in yeast reflects a true binding of CMV to Mpcp4 within vector stylets remains to be demonstrated, and the role of this protein in CMV transmission has not yet been assessed; however, the very recent identification of Mpcp4 in the acrostyle (named Stylin-01 by Webster and co-workers [15^••^], see below) supports this CuP as a CMV receptor candidate. In the second study, a peptide array approach, based on results showing the presence of RR-2 proteins in the acrostyle [40^•^], was developed to characterize CuP-virus interactions [25]. Two consensus sequences were deduced from the pattern of hybridization of CMV onto the array, one within the RR-2 chitin-binding domain, the other being a sequence frequently found in RR-2 proteins, likely exposed on the acrostyle surface [25]. These two studies revealed that CMV interacts with both RR-1 proteins in yeast, and with RR-2 peptides *in vitro* (Table 1, Figure 1). As these two types of CuPs are part of the acrostyle [15^••^], we hypothesize that they both play a role in virus retention. However, as already mentioned for ZYMV, binding of CMV in the common canal lacks direct evidence [37], and additional biochemical and functional validation are needed to determine the role of these CuPs in CMV transmission.

A recent study has made great strides towards noncirculative virus receptor identification [15^••^]. By immunolabeling with a series of antibodies specific to annotated CuPs from the CPR family, two highly homologous RR-1 proteins, Stylin-01 and Stylin-02, were detected at the tip of aphid stylets [15^••^]. A peptide corresponding to the C-terminus of Stylin-01 and Stylin-02 was shown to evenly cover the surface of the acrostyle, and to overlap with CaMV retention sites. Two series of experiments support Stylin-01 as a receptor candidate for CaMV (Table 1, Figure 1): firstly, CaMV helper protein P2 and the antibody targeting the surface-exposed peptide compete for binding to the acrostyle, and secondly, silencing Stylin-01 in the vector species *M. persicae* via RNA-mediated interference resulted in a 40% decrease in CaMV transmission efficiency.

To date, the only CuPs identified in insect stylets are proteins from the CPR family [15^••^,^25^,40^•^]. Both RR-1 and RR-2 proteins have been detected at the tip of maxillary stylets, displaying domains at the interface in direct contact with contaminated phloem sap. And both are involved in virus–vector interactions. One of the RR-1 proteins is already clearly involved in CaMV transmission, and some results suggest that it might also be important for the transmission of cucumoviruses. However, current knowledge does not distinguish if only a single CPR protein is required for binding all noncirculative viruses, which includes hundreds of virus species, or if several CuPs can act as receptors of noncirculative viruses within insect stylets.

### Role of cuticular proteins in circulative plant virus transmission

Since circulative transmission entails crossing several vector tissues and travelling within the insect body, various vector molecules are likely to interact with circulative viruses throughout their journey to, firstly, promote virus entry into, and release from, insect tissues, secondly, facilitate virus movement, thirdly, protect viruses from degradation within the insect hemolymph, and lastly, play a role in virus replication in the case of propagative viruses. In addition to classical methods such as YTH screenings, current attempts to characterize insect partners of circulative viruses often rely on the development of high-throughput approaches such as proteomic and transcriptomic analyses able to detect changes in protein abundance or genes differentially expressed between viruliferous or healthy insects. Generally, these studies generate listings with many protein candidates, including some CuPs [13^••^,18^••^,^19^, ^20^, ^21^,^23^,^26^,^46^, ^47^, ^48^]. These listings are important resources of potential virus putative partners. However, the major issue will be to confirm virus–CuP interactions and to validate the role of these candidates in virus transmission.

In a pioneering study, Seddas *et al.* [16] searched for aphid proteins that interact with a polerovirus, the beet western yellows virus (BWYV, currently known as turnip yellows virus or TuYV [49]), and that could be involved in virus transmission. Using wt viruses or derived non-transmissible mutants as overlays, a few aphid proteins of *M. persicae* were revealed by far-western blot analyses, and a CuP of 33 kDa was identified from 2-dimensional gel electrophoresis and mass spectrometry analysis (Table 1). This CuP was shown to interact strongly with wt BWYV, but not with the mutant lacking the readthrough (RT) domain, suggesting an interaction through the RT protein (Figure 1) — a protein strictly required for aphid transmission [50] and shown to play a role in virus movement [51]. At that time, as it was unexpected to find an interaction between a circulative virus and a CuP, the authors concluded that this aphid protein might not play a role in the BWYV transmission mechanism. However, a plausible involvement of CuPs in polerovirus translocation in the gut was later suggested with the identification of several aphid CuPs that correlated with a transmission efficient phenotype and cereal yellow dwarf virus-RPV (CYDV-RPV) movement through the insect gut [18^••^]. Amongst the aphid proteins predicted to facilitate virus entry at the gut barrier, two CuPs were detected: one RR-2 and CPG12 (gi:193647865 and gi:193706873, respectively) (Table 1, Figure 1). Additionally, two other RR-2 CuPs (gi:193647875, gi:193582403) were upregulated both in competent and hindgut-refractive genotypes (Table 1), precluding any clear conclusion on their role in virus translocation [18^••^]. In a third recent study [20], interactions between barley yellow dwarf virus BYDV-GPV, a member of the *Luteoviridae* family, and CuPs from its aphid vector *Rhopalosipum padi* were also reported. The two insect partners, which are RR-2 proteins, were identified using two approaches: firstly, differential proteomics analyses between healthy and viruliferous *R. padi* (Table 1, Figure 1), and secondly, YTH using viral RT protein as bait (Table 1, Figure 1). Interestingly, this second RR-2 protein, called *cuticular protein 5 precursor*, is highly homologous with CYDV-RPV partners (gi:193582403, gi:193647865, [18^••^]). Although additional experiments are required to determine whether CuPs play a role in luteovirids transmission by facilitating virus entry at the gut level or at another step of the transmission process, taken together, these results stress the importance of characterizing luteovirids–CuPs interactions in future work.

A role for a CuP in circulative virus transmission was formally demonstrated for rice stipe virus (RSV) [13^••^] — a tenuivirus transmitted mostly by the planthopper *Laodelphax striatellus* in a propagative manner [52]. The authors provided the only direct experimental evidence thus far of an interaction between a CuP and a circulative virus within an insect vector. CPR1, an RR-1 protein, was identified by YTH screening of a cDNA library using the viral nucleocapsid protein pc3 (N) as bait (Table 1). A strong interaction between CPR1 and pc3 proteins was confirmed *in vivo* by a co-immunoprecipitation assay. Moreover, the two proteins co-localized in insect cell culture, and in hemocytes isolated from the hemolymph of viruliferous insects (Figure 1), whereas silencing CPR1 in *L. striatellus* resulted in a 57% decrease in RSV transmission capacity. The authors proposed that CPR1 could bind virus particles in the hemolymph and hence assist viral movement towards the salivary glands [13^••^]. Two additional CuPs were shown to interact with RSV nucleocapsid protein N (pc3) in another, YTH-independent, screening [26]. These CuPs belong to the CPR (RR-2) and Tweedle protein families. It will be interesting to determine if RSV can interact with several CuPs within its vector, and if several CuPs are required for successful transmission. However, interactions of these novel candidates have not been confirmed in the vector, and their role in RSV transmission remain to be investigated thoroughly [26].

## Role of cuticular proteins in arbovirus transmission

Interestingly, CuPs have also been reported to potentially interact with arboviruses, but the precise role of CuPs in arboviruses infection is not well documented. A deregulation of CuP gene expression in insects upon infection with animal viruses has been reported [53,54^••^,^55^]. In a more extended study, Colpitts *et al.* [54^••^] characterized a mosquito-borne flavivirus–CuP interaction. The authors investigated the role of a RR-2 pupal CuP, whose transcripts were downregulated in *Aedes aegypti* pupae upon infection by West Nile (WNV), dengue (DENV) or yellow fever (YFV) viruses. Overexpression of this CuP gene in mosquito cells or in live mosquitoes inhibited WNV infection. Furthermore, this pupal CuP interacted with the envelope of WNV, DENV and the capsid of YFV, which might impede viral entry to host cells. More importantly, pre-incubation of WNV with pupal CuP prevented lethal WNV encephalitis in mice. This work highlights once again the key role of a CuP from the CPR family in virus transmission through a direct virus–vector interaction. However, in contrast to vectored plant viruses for which CuPs have been shown to promote virus transmission, chitin-binding proteins were instead proposed to protect the insect from viral infection by preventing virus–receptor interactions, or by strengthening natural barriers to pathogen infection.

## Conclusions

Cuticular proteins now feature prominently on the shortlist of insect molecules demonstrated to play a key role in plant virus transmission [12^••^,13^••^,14^••^,15^••^]. Recent literature has revealed that CuPs—the most obvious candidates as receptors for noncirculative viruses — also interact with plant circulative viruses. Current knowledge indicates that insect CuPs may facilitate their entry at the gut level, and assist virus particles in the hemolymph.

Almost all the CuPs identified so far in plant virus–vector interaction studies belong to the large CPR family. More precisely, while the role of RR-2 proteins in virus transmission remains to be determined, RR-1 proteins are definitely associated with circulative and noncirculative virus transmission. Future research should help define whether additional CuPs also participate in the transmission process.

The data presented in this review highlight the interest in strengthening further efforts to characterize insect CuPs and their interactions with plant viruses. In the future, we can count on growing interest from virologists to characterize CuPs, as novel candidates in the search for innovative viral control strategies.

## Conflict of interest statement

Nothing declared.

## References and recommended reading

Papers of particular interest, published within the period of review, have been highlighted as

• of special interest
•• of outstanding interest
